# Investigating the Potential and Pitfalls of EV-Encapsulated MicroRNAs as Circulating Biomarkers of Breast Cancer

**DOI:** 10.3390/cells9010141

**Published:** 2020-01-07

**Authors:** Brian M. Moloney, Katie E. Gilligan, Doireann P. Joyce, Clodagh P. O’Neill, Killian P. O’Brien, Sonja Khan, Claire L. Glynn, Ronan M. Waldron, Ciarán M. Maguire, Emma Holian, Erin Naughton, Mohamed Elhadi, Andrea B. Grealish, Carmel Malone, Emma McDermott, Peter Dockery, Thomas Ritter, Adriele Prina-Mello, Michael J. Kerin, Róisín M. Dwyer

**Affiliations:** 1 Discipline of Surgery, Lambe Institute for Translational Research, School of Medicine, National University of Ireland Galway, Galway H91 YR71, Ireland; 2 Laboratory for Biological Characterisation of Advanced Materials (LBCAM) and Advanced Materials and Bioengineering Research (AMBER) Centre, Trinity Translational Medicine Institute, Trinity College Dublin, James Street, Dublin D08 W9RT, Ireland; 3 School of Mathematics, Statistics and Applied Mathematics, National University of Ireland Galway, Galway H91 YR71, Ireland; 4 Centre for Microscopy and Imaging, Discipline of Anatomy, School of Medicine, National University of Ireland Galway, Galway H91 YR71, Ireland; 5 Regenerative Medicine Institute, School of Medicine, College of Medicine, Nursing and Health Sciences, National University of Ireland Galway, Galway H91 YR71, Ireland

**Keywords:** breast cancer, extracellular vesicles, exosomes, microRNA, biomarker, EV characterization

## Abstract

Extracellular vesicles (EVs) shuttle microRNA (miRNA) throughout the circulation and are believed to represent a fingerprint of the releasing cell. We isolated and characterized serum EVs of breast tumour-bearing animals, breast cancer (BC) patients, and healthy controls. EVs were characterized using transmission electron microscopy (TEM), protein quantification, western blotting, and nanoparticle tracking analysis (NTA). Absolute quantitative (AQ)-PCR was employed to analyse EV-miR-451a expression. Isolated EVs had the appropriate morphology and size. Patient sera contained significantly more EVs than did healthy controls. In tumour-bearing animals, a correlation between serum EV number and tumour burden was observed. There was no significant relationship between EV protein yield and EV quantity determined by NTA, highlighting the requirement for direct quantification. Using AQ-PCR to relate miRNA copy number to EV yield, a significant increase in miRNA-451a copies/EV was detected in BC patient sera, suggesting potential as a novel biomarker of breast cancer.

## 1. Introduction

Initially deemed a mechanism of cellular waste disposal, extracellular vesicles (EVs) secreted by cells are now known to encapsulate a variety of biomolecules thought to be reflective of the cell from which they are released [1]. As such, they hold significant potential as circulating biomarkers of disease, with proposed applications in cancer diagnosis, prognostication, and prediction or monitoring of response to therapy [1,2,3,4,5,6,7]. However, rapid expansion in the field, as evidenced by a surge in publications, is not without challenges. There has been a significant amount of conflicting evidence in the literature regarding EVs with respect to nomenclature, isolation, characterization, quantification, and transparency of reporting standards [8,9,10,11,12]. A myriad of EV purification and characterization methods are employed by different research groups, often with poor reporting of key experimental parameters. This issue was explored in detail by the EV-TRACK Consortium (2017) which paved the way for development of the “EV-METRIC”, with the aim of improving completeness of reporting on study methodology going forward [11]. More recently, a further update was provided through publication of the Minimal Information for Studies of Extracellular Vesicles (MISEV) 2018 guidelines [12]. There is a move to referring to exosomes and other vesicles simply as EVs, recognizing the heterogeneity of all isolates, while providing detailed descriptions of isolation methods and specific characterization criteria. The universal adoption of this approach should increase transparency of reporting and support reliable comparison of data generated by different groups, supporting progress to clinical application.

EVs encapsulate a plethora of potentially clinically relevant biomolecules, including microRNAs (miRNAs) [1], which have been at the forefront of breast cancer research in recent decades, with innumerable studies attempting to elucidate biomarker potential [13,14,15]. Despite initial promise, circulating miRNAs have yet to be implemented in a clinical setting. The reason for this is multifactorial and includes broad variations in starting materials (whole blood, serum, or plasma) and extraction methods, and inconsistent use of endogenous controls [13]. As a result of this, the body of research on cancer-related miRNAs is contrasting, with conflicting results reported for the same miRNA being commonplace, even when evaluated in an identical disease setting [13]. Limiting the focus of interest to the miRNA contained in the EV fraction of blood alone may help to overcome these challenges. It is imperative, however, that we remain cognizant of these pitfalls, and avoid the initial mistakes made in the field of miRNA-oriented biomarker discovery and validation, when attempting to expound the clinical relevance of EV-encapsulated miRNAs (EV-miRs).

There have been a number of initial studies investigating circulating EV-miRNAs as biomarkers of breast cancer in recent years [5,16,17,18,19,20,21]. Although limited by control sample size, one study reported elevation of miRNA-101 and miRNA-372 in serum-derived EVs of patients with breast cancer (*n* = 50) compared to healthy controls (*n* = 12) [5]. The use of EV-miRNAs as a prognostic marker for metastatic progression in breast cancer was investigated by Zhou et al. [16], employing serum EVs in a murine model of breast cancer and in 38 patients. Significantly elevated EV-miR-105 levels were detected in patients who went on to develop distant metastases (*n =* 16) compared to those who did not (*n =* 22). In these studies, like many published at the time, either no quantification or indirect quantification of EVs using a protein assay was employed, and so, variable amounts of EV template may have been inadvertently included in each group.

Analysis of plasma EV-miRNAs in breast cancer has also been performed using patient derived xenograft (PDX) murine models, breast cancer patients, and healthy controls (*n =* 16 each) [17]. EV-miR-21 and miR-1246 were found to be significantly elevated in the plasma of patients with breast cancer compared to healthy controls. For normalization of RT-PCR data, a synthetic *Caenorhabditis elegans* miR-54 (cel-miR-54) RNA oligonucleotide was used as a spike-in control, as no robust endogenous controls for exosome/EV-miRNAs exist. While one study reported the use of plasma EV-miR-16 as an endogenous control [21], another reported dysregulation of plasma EV-miR-16 in patients with breast cancer [18]. In the latter study, miR-484 was employed as an endogenous control, along with synthetic cel-miR-39, as miR-484 showed the smallest variation between healthy controls and patient samples. However, there was no apparent standardization of loading material, and following isolation of EVs using a commercial kit, samples were characterized only by the presence of CD63 and absence of Ago2 (usually associated with cell free miRNA) by western blot [18]. As a result, data may be impacted through analysis of differing yields of EVs in patient samples, thereby impacting the levels of miRNAs detected. The studies outlined provide an important proof-of-principle, despite their limitations in size and scope.

In the largest study published to date, Stevic et al. [19] analysed the microRNA profiles of serum EVs from 435 breast cancer patients. Only 20 healthy control individuals were included in the study, with the focus being on comparison of patients with Her2 amplified subtype (Her2+, *n =* 211) and those with triple negative breast cancer (TNBC, *n =* 224). Microarray data was normalized using miR-92a and miR-484, which were identified as relatively stable across arrays. However, there was no standardization of loading material, with RNA isolated from total EV yields and resuspended in the same volume for analysis. Indirect quantification of EVs in a subset of samples employing a CD63 ELISA (Her2+ (*n =* 78), TNBC (*n =* 40), and healthy controls (*n =* 10)) revealed a significant increase in EVs in both breast cancer groups compared to controls, with a higher (non-significant) level also reported in Her2+ compared to TNBC patients [19]. This is likely to have impacted the levels of miRNA detected. As our knowledge of EV characteristics and cargo continues to evolve, it is becoming increasingly important to standardize effective methods for analysis of the biomolecules within, and analysis of data generated.

EV-specific miR-451a, analysed further in the current study, has previously been demonstrated to be elevated in the circulation of patients with non-small cell lung cancer [22] and to have potential applications in prognostication of patients with pancreatic ductal adenocarcinoma [23]. In this study we describe isolation of cell secreted EVs and circulating EVs in animal models and patients with breast cancer. This is followed by detailed EV characterisation. EV-miR-451a was quantified in the circulation of tumour-bearing animals, and breast cancer patients (*n =* 67) were compared to healthy control (*n =* 44) individuals. The HCC-1954 model is a HER2 amplified human breast cancer cell line, so was implanted into immunocompromised, athymic Balb/c mice. In the absence of an established EV-miRNA endogenous control or robust method for data normalization, absolute quantification of microRNA copy number is related to total EV number.

## 2. Materials and Methods

### 2.1. Culture of Breast Cancer Cell Lines

HCC-1954-luc breast cancer cells were routinely maintained in RPMI-160. Media was supplemented with 10% FBS and 100 IU/mL penicillin G/100 µg/mL streptomycin sulphate (Pen/Strep). The cells were originally purchased from LGC limited and were authenticated every two years using single tandem repeat (STR) analysis. For bioluminescent in vivo imaging, HCC-1954-luc cells were previously transduced with lentivirus expressing a red-shifted *Luciola Italica* luciferase transgene, under the control of the Ubiquitin C (UbC) promoter (RediFect Red-FLUC-Puromycin Lentiviral particles, Perkin Elmer Maryland, USA) [24].

### 2.2. Patient Samples and Ethics

All experimental procedures involving sera from human participants were approved by the Clinical Research Ethics Committee (University College Hospital, Galway). Written informed consent was obtained from each patient and all clinical investigation was performed according to the principles expressed in the Declaration of Helsinki [25]. A total of 111 female participants were enrolled for this study. This included 67 patients with breast cancer with a mean age of 55 (range 28–84) and 44 healthy volunteers with a mean age of 50 (range 23–69; Table 1). Healthy control volunteers had no family history or a personal medical history of breast cancer. Volunteers deemed to have co-morbidities requiring active treatment were excluded from the study. Of the patients with breast cancer, 54 had the primary tumour in situ, while 13 had breast cancer metastasis following initial resection. Disease characteristics are detailed in Table 1. All serum samples were collected in Vacutainer Serum Separator Tubes II (Becton Dickinson), allowed to clot for 30 min, and centrifuged at 805× *g* at 4 °C for 10 min. Serum was then stored at −80 °C until required for EV isolation.

### 2.3. In Vivo Breast Cancer Model

Ethical approval for In Vivo studies was granted from the Animal Care Research Ethics Committee at the National University of Ireland Galway. Project authorisation was obtained from the Health Products Regulatory Authority (HPRA) of Ireland.

Twenty-two female, athymic BALB/c nude mice (Charles River Laboratories Ltd. Kent, UK) aged 6–8 weeks were used in the study. All mice under anaesthetic (5% Isoflurane) received a mammary fat pad (MFP, second thoracic) injection of 3.5 × 10^6^ HCC-1954-luc cells suspended in 200 µL RPMI medium. All animals were drug naïve, had no previous procedures performed, and weighed between 18–20 g. Each animal’s environmental enrichment was monitored daily, including health and behaviour, cage conditions, food, and water. Cells were injected under sterile conditions on a surgical mount/heated stage. Disease progression was monitored using an in vivo imaging system (IVIS, PerkinElmer, Massachusetts, United States) following intraperitoneal (IP) injection of luciferin at 150 mg/kg. Approximately 6 weeks following tumour induction, animals were humanely sacrificed by cardiac puncture under isoflurane anaesthesia. Blood was harvested and serum was extracted from individual whole blood samples (not pooled) and stored at −80 °C until required for EV isolation.

### 2.4. Isolation of EVs from Human/Murine Sera

To isolate EVs from sera, 500 µL patient sera (*n =* 111) or animal sera (*n =* 22) was thawed and diluted in 12 mL PBS; that was followed by differential centrifugation at 800× *g* and 2000× *g* for 10 min each, microfiltration (0.22 µm), and ultracentrifugation (Hitachi Koki himac, micro-ultracentrifuge CS150FNX; rotor S50A-2152) at 1.1 × 10^5^× *g* for 120 min. EVs isolated from sera were re-suspended in sterile PBS and aliquoted for subsequent RNA isolation, protein analysis, NTA analysis, or TEM. Protein lysis solution (1% Triton X-100 in 20 mM Hepes, 2 mM EDTA, 150 mM NaCl, 10 mM sodium fluoride, 100X Protease Inhibitor Cocktail, and 2 mM sodium orthovanadate) was added to the samples destined for protein analysis. EVs were stored at −80 °C until required.

### 2.5. Characterisation of Extracellular Vesicles

To confirm the presence of EVs, morphological examination of fixed EVs embedded in resin was performed as previously described [26]. Briefly, a primary fixative (2% glutaraldehyde, 2% paraformaldehyde in a 0.1 M sodium cacodylate/HCL buffer, pH 7.2) was added to samples prior to ultracentrifugation, and following isolation, EVs were immersed in a secondary fixative (1% osmium tetroxide), dehydrated in alcohol, and embedded in resin. Resin slices were then loaded onto a copper grid, stained, and viewed using an Hitachi H7000 transmission electron microscope [26].

Western blots were performed, targeting the EV-associated proteins CD81 (Abcam ab79559), CD82 (Abcam ab66400) and CD63 (Abcam ab68418). Protein concentration was determined by microBCA Assay (Pierce™, Thermo Fisher Scientific, Massachusetts, USA) according to manufacturer’s instructions. Protein samples (10 µg) were denatured for 10 min at 70 °C, which was followed by separation on a pre-cast Mini-PROTEAN^®^ TGX™ Gel (Bio-Rad) for 60 min at 100 V. Protein molecular weight standards (20–220 kDa) were run simultaneously on each gel, followed by transfer to a nitrocellulose membrane. Once blots were blocked (5% milk in TBS-T for 1 h), each membrane was probed with an antibody targeting CD81 (1:1000 dilution, 1.5 h, RT), CD82, or CD63 (both at 1:1000 dilution; overnight, 4 °C) diluted in 0.1% milk in TBS-T. Following a series of washing steps, a solution of secondary antibody (CD81—1:10,000; rabbit anti-mouse IgG HRP; Abcam ab6728; CD82 and CD63—1:3000; goat anti-rabbit IgG HRP; Abcam ab6721) was added to each membrane. Clarity™ Western ECL (Bio-Rad Laboratories, Maryland, USA) chemiluminescent substrate solution was applied to each membrane and blots were visualized using the Gel Doc™ XR+ and ChemiDoc™ XRS + Systems with Image Lab™ Software (Bio-Rad, version 5.2.1).

Nanoparticle tracking analysis (NTA) (NanoSight NS500, Malvern Panalytical, UK) was used to analyse EV particle size distribution and concentration using a 405 nm laser source and EMCCD camera, running NTA software version 3.2 using optimised and validated protocols [27]. EV samples were diluted in PBS certified as particle-free by NTA (<3 particles per frame visible). Instrument calibration was verified daily using 100 nm polystyrene latex calibration nanoparticles (Malvern Panalytical). A total of five 60 s videos were recorded for each sample [27]. Total EVs per microliter were determined, along with quantification of those within 30–150 nm in size, representing the small EV (sEV) fraction. Spearman’s rank correlation coefficient was calculated to assess the relationship between total protein yield determined by microBCA assay, and number of EVs (total, and 30–150 nm size) was directly quantified by NTA.

### 2.6. RNA Extraction and Absolute Quantitative (AQ)-PCR Analysis

RNA was extracted from EV isolates (animal and patient sera) using the MagNA Pure Isolation (Roche) extraction process as per manufacturer’s instructions, and stored at −80 °C. MicroRNA (miR-451a) expression analysis was performed using TaqMan^®^ assays and Universal Mastermix (Applied Biosystems) [28]. An inter-assay control was employed on each plate, and all samples were analysed in triplicate (standard deviation <0.3 required). Absolute quantification was used to determine the number of copies of miR-451a relative to a standard curve of known concentrations of synthetic miR-451a. Briefly, synthetic miRNA-451a (Applied Biosystems) was reconstituted in T/E buffer (10 mM Tris-HCl, 1 mM disodium EDTA, pH 8.0) to generate a 2.38 × 10^−4^ mol stock solution. A working solution of 1 × 10^−5^ mol was produced and reverse transcription performed, as described previously [28]. Serial dilutions were prepared of standards ranging from 1 × 10^−8^ to 1 × 10^−14^ M. The comparative cycle threshold was used to calculate the quantity of miR-451a in samples relative to the standard curve generated [29,30]. The resultant value, obtained in moles, was converted to particles by multiplication through Avogadro’s constant (6.03 × 10^23^ particles) [31].

### 2.7. Statistical Analysis

Continuous variables of interest are summarised numerically by means (with SEMs) and are graphically represented using boxplots. Shapiro–Wilk’s W test was employed to test the assumption of normality. Levene’s test was used to assess the equality of variances for a variable calculated for two or more groups. Comparisons of continuous parametric variables were performed using a Student’s *t*-test or ANOVA. Comparisons of non-parametric variables were performed using the Mann–Whitney test. The degree of relationship between pairs of response variables was assessed using the Pearson or Spearman correlation coefficient, as appropriate. A two-tailed *p* value of less than 0.05 indicated statistical significance. All analyses were performed using SPSS Version 22 (IBM Corporation, New York, NY, USA) and Minitab 17 (Minitab, Inc., State College, PA, USA).

## 3. Results

### 3.1. Routine Characterisation of Isolated EVs

**Figure 1:**
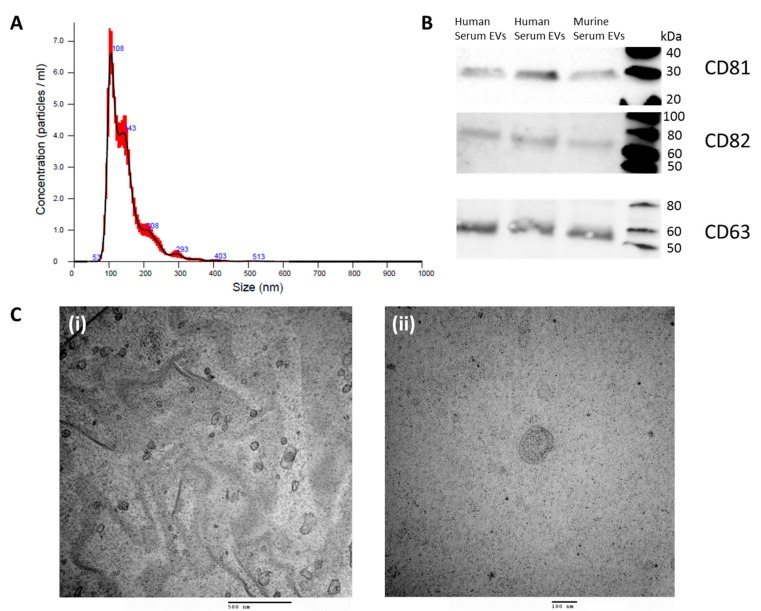
Serum extracellular vesicle characterisation: (**A**) Nanoparticle tracking analysis—the mean size distribution and concentration (1 × 10^9^) of the particles. The line represents the average of five readings with red shading showing range across readings; (**B**) western blot analysis confirming the detection of CD81 (27–30 kDa), CD82 (60 kDa), and CD63 (50–60 kDa) at the appropriate band sizes. (**Ci**) TEM wide field view image (60,000×) and (**Cii**) close field view (120,000×) demonstrating EVs with a round morphology and lipid bilayer.

To confirm the presence and assess the quantity of EVs isolated from sera, NTA, western blotting, and TEM were routinely performed, with representative images shown in Figure 1. Isolated EVs were characterised by NTA, which determined the number and size distribution, with the majority of vesicles falling within the size range of small EVs (sEVs), at 30–150 nm in size (Figure 1A). Western blot analysis was performed, which confirmed that isolates expressed the EV-associated proteins CD81 (27–30 kDa), CD82 (60 kDa, n-glycosylated), and CD63 (50–60 kDa) at the appropriate band sizes (Figure 1B). EVs were visualized using TEM in both wide field (60,000×), showing multiple EVs of similar size and close field (120,000×) views, showing details of the lipid bilayer (Figure 1(Ci,Cii)). Vesicles of rounded morphology measuring 30–150 nm in diameter consistent with reported characteristics of sEVs were observed.

### 3.2. Quantifying EVs in Human Serum

The total number of EV particles isolated from 500 µL sera as determined by NTA, ranged from 3.42 × 10^8^ to 8.90 × 10^10^/mL across all human serum samples (cancers and healthy controls, Table 2). The mean total number of EV particles was significantly higher in patients with breast cancer when compared to control serum samples (BrCa 1.85 × 10^10^ ± 1.99 × 10^9^/mL versus control 1.18 × 10^10^ ± 1.42 × 10^9^/mL, Mann–Whitney U = 927.5, *p* = 0.001).

Data relating to EVs in the size range associated with sEVs (30–150 nm) were then analysed. The number of EV particles of this size ranged from 1.26 × 10^8^ to 5.73 × 10^10^/mL of serum for all samples. Similar to the analysis involving total EV particles, there was a significant increase of EV particles within this size range between the breast cancer patients and healthy volunteer individuals (9.30 × 10^9^ ± 1.24 × 10^9^/mL versus 6.29 × 10^9^ ± 1.01 × 10^9^/mL, U = 1110.0, *p* = 0.028). Patients with breast cancer were further subdivided based on disease characteristics (outlined in Table 1) to determine whether there was any relationship between EV number and other disease characteristics, such as disease stage. No significant relationship was observed (e.g., disease stage UICC classification *p* = 0.869, Figure S1A). Although the highest number of EVs was detected in patients with metastatic disease (*n =* 13), this was not significantly higher than those with primary disease (*n =* 54), with both groups having a significant increase over healthy controls (Figure S1B).

**Figure 2:**
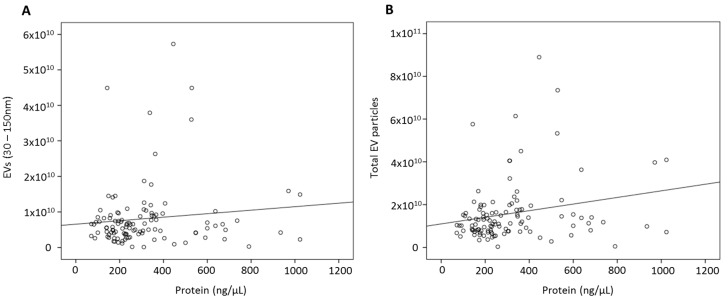
Investigation of relationship between protein yield and number of EV particles quantified by NTA: No relationship was detected between (**A**) protein yield and small EV particles (30–150 nm, rho = 0.092, *p* = 0.339) with only a mild correlation between protein yield and total number of EV particles (**B**) detected (rho = 0.216, *p* = 0.027) in human serum samples.

In relation to protein yield, despite a standardised volume of starting material, there was a wide range of protein quantity present across all human serum EV samples (*n =* 111, range 70.6–1023.3 ng/µL), with no significant difference in sera from patients with breast cancer (345.2 ± 25.9 ng/µL) when compared to their healthy counterparts (268.1 ± 28.2 ng/µL, *p =* 0.053). In the past, protein yield has been widely used as a surrogate indicator of sEV quantity; however, as demonstrated in Figure 2A, no relationship between sample total protein yield and number of exosomal EVs was detected (Spearman’s rho = 0.092, *p =* 0.339), with only a weak correlation with total EV yield detected (rho = 0.216, *p =* 0.027, Figure 2B).

### 3.3. Detection of EV-miR451a in a Breast Tumour Bearing Murine Model

**Figure 3:**
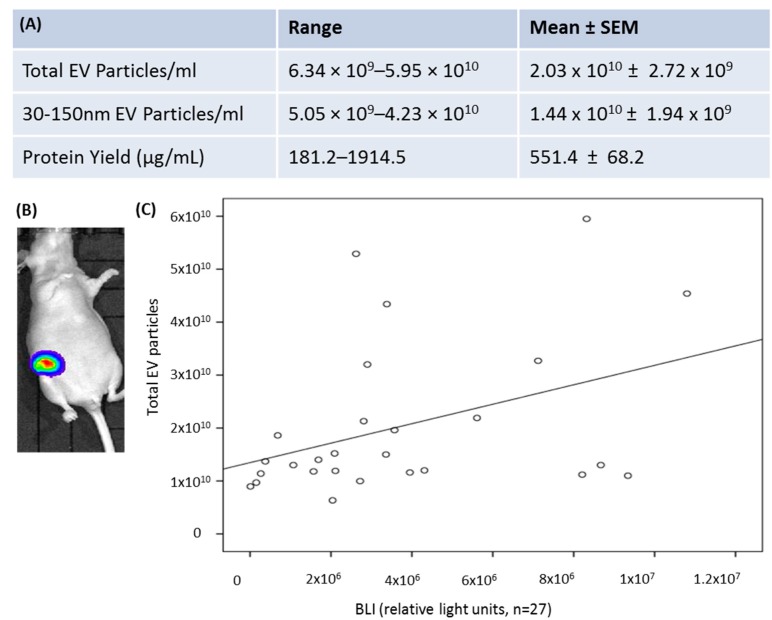
(**A**) EV yield as determined by nanoparticle tacking analysis, and protein yield, as detected by microBCA assay from the sera of HCC-1954-luc tumour-bearing mice. (**B**) Sample bioluminescent IVIS image from a mouse bearing mammary fat pad tumour; (**C**) relationship between BLI (relative light units) and number of circulating EVs.

EV-miR-451a was confirmed to be secreted by HCC-1954-luc cells in vitro, and then investigated in the serum EVs of HCC-1954-luc tumour bearing animals. Animals were treatment naïve prior to blood sampling, with no adverse events seen. As for the patient samples, murine serum EVs were quantified using NTA and protein yield was determined for the purposes of western blot analysis (Figure 3A). Bioluminescent imaging (BLI) using IVIS provided quantitative data on tumour burden (relative light units, RLU, sample image shown in Figure 3B).

MiR-451a was detected in all murine serum EV samples analysed (*n =* 22). Calculation of miR-451a copy number was determined by absolute quantification. A standard curve for 10^−8^ to 1 × 10^−14^ mol dilutions of synthetic miR-451a was reproduced and a linear Equation (1) was accepted (Figure S2A). An abundance of miRNA 451a was detected in the circulating EV particles of all breast cancer bearing murine models analysed (4.84 × 10^−14^–7.52 × 10^−13^ mol/mL). Applying Avogadro’s constant, the miR-451a copy number was calculated to range from 2.92 × 10^10^ to 4.54 × 10^11^ copies/mL (1 mol = 6.02 × 10^23^ particles). Although a small group (*n =* 22), preliminary analysis revealed a moderate correlation between serum EV number and tumour burden measured by BLI (rho = 0.386, *p* = 0.047, Figure 3C).

y = −3.70 X − 14.80.(1)

### 3.4. EV-Encapsulated MiR-451a in Sera of Breast Cancer Patients and Healthy Controls

**Figure 4:**
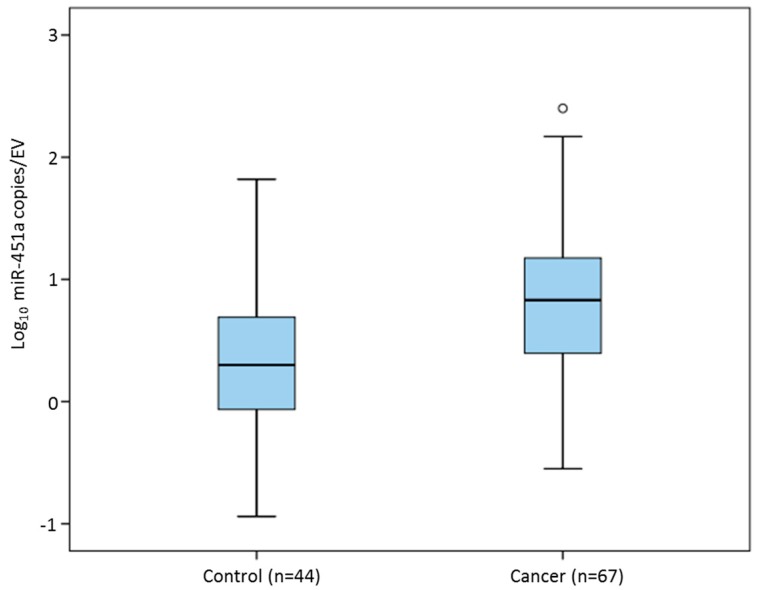
Number of miRNA-451a copies per EV particle in sera of breast cancer patients (*n =* 67) compared to healthy control individuals (*n =* 44, *p* = 0.029), o represents outlier

A standard curve for 10^−8^ to 1 × 10^−14^ mol dilutions of synthetic miR-451a was reproduced and an average linear equation (Equation (2)) accepted (Figure S2B). After determining total number of miRNA-451a copies per sample by multiplication by Avogadro’s constant, total number of copies of miRNA-451a was expressed relative to the number of EVs in each sample. The mean number of copies of miRNA per EV ranged from 0.12 to 249.10 copies/EV. The mean number of miR-451a copies per EV was significantly greater in the patients with breast cancer in comparison to the healthy cohort (15.8 ± 4.4 versus 5.5 ± 1.7 copies, *p =* 0.029). A logarithm of the means was employed in Figure 4 to demonstrate this comparison. When expressed relative to the number of exosomal EVs (30–150 nm), a similar outcome was observed (38.60 ± 10.85 versus 11.63 ± 3.10 copies/EV, *p =* 0.019). When the breast cancer cohort were investigated in isolation, no relationship between disease stage and the mean number of miR-451a copies/EV was observed (5.58–24.44 copies/EV, ANOVA *p =* 0.549).

y = −3.24 X − 11.31.(2)

## 4. Discussion

Inter-laboratory reproducibility is crucial for EV-miRNAs to reach their potential as biomarkers or therapeutics. The data presented demonstrates that the previously widely used approach of indirectly quantifying EVs based on protein yield, bears no significant relationship with the number of EVs present in a sample when quantified directly using NTA. This was verified across patient serum samples and murine samples. Samples were isolated in two different laboratories to ensure sample collection method nor personnel were responsible, and NTA analysis was performed at a tertiary site, using ISO certified equipment. These findings have important implications for in vitro, in vivo, and clinical studies investigating the biomarker or therapeutic potentials of EVs. If samples are compared based on standardized protein yield, investigators may in fact be analysing the target (miRNA, protein, mRNA, etc.) in samples containing significantly different amounts of EV template. Therefore, elevated levels may be due to unintentional analysis of elevated EVs, rather than a clinically relevant increase in the analyte. Similarly, when testing the therapeutic potential of an EV population, it is imperative that equal amounts of EVs from different cell sources or with different modifications are loaded to ensure standardized comparison. This will also be critical for reproducibility in clinical trials. As the range of platforms available for particle analysis has increased due to market demand, with relative decreases in associated costs, access, and availability, research should be more widespread, allowing more teams to align with the MISEV guidelines in this area [12].

The choice of starting material (serum or plasma) tends to be most influenced by whichever sample type is routinely collected and accessible in the host lab. There is some concern that the clotting step in serum isolation will significantly deplete the EV fraction; however, there have been many studies successfully employing sera in breast and other cancer settings [1,4,5,16,32]. Equally there is concern that EVs isolated from plasma are coated with proteins and lipids likely to cause their aggregation and a potential loss upon centrifugation [33]. We have found serum to be a robust source of EVmiRs, as demonstrated in NTA analysis outlined previously, in agreement with other groups who reported plasma or sera as equally good sources of circulating EVs based on the recovery, purity, morphology, and biological function [33]. There is no doubt that all sample sources present their own challenges, and robust characterisation of isolated EVs is an absolute requirement.

The quantity of EVs released into circulation is a consequence of the site of origin, and these particles can envelope an extensive plethora of proteomic content. Taking ovarian cancer as an example, over 2000 species of protein have been identified from tumour-derived EVs, including many involved in disease progression and metastasis, such as membrane proteins, tetraspanins, and enzymes [34]. Serum samples were derived from cancer patients with different stages of disease and a range of disease burden as indicated in Table 1. Healthy control volunteers had no family history or a personal medical history of breast cancer; however, women of a variety of ages and backgrounds were included. While all patients satisfied the inclusion criteria for the study, factors that can impact protein content, such as excessive amounts of cells in the standardised starting volume, increased platelet activation, elevated immune and inflammatory response, and high concentrations of procoagulant and angiogenic agents were not accounted for [35]. Within the human sera analysed, a significantly higher quantity of EVs was observed in the cancer patients, compared to healthy controls. It is conceivable that a false increase in candidate miRNAs could then have been detected based on elevated EV template, highlighting the need for endogenous controls. Preliminary analysis also revealed a moderate correlation between tumour burden measured by BLI and the number of circulating EVs in murine sera (*n =* 22, rho = 0.386, *p =* 0.047). Cancer cells are believed to release more EVs into the circulation than normal cells [32,36,37], although it is important to note that the EVs come from a variety of cell sources and are representative of the host as much as the cancer. While direct quantification of EVs is important, robust and reliable protocols are also required, as inter-user variation has been shown to produce inaccurate results for both size and concentration measurements. This can be as high as 20% for size and 170% for concentration [38,39]. While NTA accuracy can also be hampered by the presence of co-purified EV contaminants, such as protein complexes and lipoproteins, this method does offer a means of direct quantification of EV number, which undoubtedly provides an improvement on indirect approaches [40].

EV quantification methods recently employed include NTA, tunable resistive pulse sensing (TRPS), vesicle flow cytometry, surface plasmon resonance, and electron microscopy [41]. While each have their merits, concordance across all must be questioned. A previously mentioned study reported elevated miR-1246 in plasma of a small cohort of breast cancer patients compared to healthy controls (*n =* 16 each) [17]. Zhai et al. [20] targeted the same miRNA in plasma using a novel, nucleic-acid-functionalized Au nanoflare probe. The probe enters EVs in plasma to generate a fluorescent signal by specifically targeting miR-1246, and was reported to discriminate between breast cancer (*n =* 46) and healthy control samples (*n =* 28) with 100% sensitivity and 92.9% specificity [20]. In the current study using miR-451a as an example, absolute quantification of the number of copies of the miRNA was performed and expressed relative to the number of EVs in each sample, as determined by NTA. A significant increase in miRNA-451a copies/EV was detected in sera of breast cancer patients (*n =* 67) compared to healthy controls (*n =* 44, *p =* 0.029), suggesting a role as a potential novel biomarker of breast cancer. While the authors do not suggest that miRNA copy numbers are evenly dispersed in each EV, this provides an approach that takes into account the number of EVs present in each sample. This could be impacted by a myriad of patient factors, including tumour burden, host immune response to disease or to therapy, and other co-morbidities, all of which will then impact the data generated.

## 5. Conclusions

It is clear that EV source, isolation, and characterization methods; data normalization and analysis; and reporting of study parameters, are heterogeneous in the published literature. If we do not address the controversies faced by this rapidly evolving field, there is a danger that the true potential of EVs may not be fully realized. It is imperative to address these issues and perform appropriately powered studies, using clinically relevant models of disease, patient samples that represent the heterogeneity of breast cancer and appropriately matched controls, taking into account the impact of standard clinical interventions, and immune response to disease. Multidisciplinary collaboration will be key to realizing the immense potential of EV-miRNAs as robust, clinically relevant biomarkers of disease.

## Figures and Tables

**Table 1:** Clinicopathological characteristics of breast cancer patients from whom serum EVs were isolated.

Healthy Volunteers	*n =* 44	Mean age (Range)	50 (23–69)
Breast Cancer Patients	*n =* 67	Mean age (Range)	55 (28–84)
Histological Invasive type		Ductal Lobular Other	54 (80.5%) 10 (14.9%) 3 (4.4%)
Epithelial Subtype		Luminal A Luminal B HER2 Basal	42 (64.1%) 9 (13.4) 10 (14.9%) 6 (8.9%)
Nodal Status		Node Positive Node Negative	32 (47.7%) 35 (52.2%)
Tumour Grade		1 2 3	4 (5.9%) 33 (49.2%) 30 (44.7%)
Stage (UICC)		I II III IV	13 (19.4%) 29 (43.3%) 11 (16.4%) 14 (20.9%)

**Table 2:** Extracellular vesicle (EV) yield as determined by nanoparticle tacking analysis and protein yield as determined by microBCA assay from the sera of patients with breast cancer and healthy control individuals.

Sample	All EV Particles/mL		30–150 nm EV Particles/mL		Protein Yield (µg/mL)	
	Range	Mean ± SEM	Range	Mean ± SEM	Range	Mean ± SEM
All Sera (*n* = 111)	3.42 × 10^8^–8.90 × 10^10^	1.59 × 10^10^ ± 1.36 × 10^9^	1.26 × 10^8^–5.73 × 10^10^	8.11 × 10^9^ ± 8.60 × 10^8^	70–1023	314 ± 20
BrCa (*n* = 67)	3.42 × 10^8^–8.90 × 10^10^	1.85 × 10^10^ ± 1.99 × 10^9^	1.26 × 10^8^–5.73 × 10^10^	9.30 × 10^9^ ± 1.24 × 10^9^	71–1023	345 ± 26
Control (*n* = 44)	3.67 × 10^9^–5.76 × 10^10^	1.18 × 10^10^ ± 1.42 × 10^9^	1.15 × 10^9^–4.49 × 10^10^	6.29 × 10^9^ ± 1.02 × 10^9^	70–935	268 ± 28

## Supplementary Materials

The following are available online at https://www.mdpi.com/2073-4409/9/1/141/s1. Figure S1: (A) Investigation of relationship between circulating EV number and disease stage (B) Circulating EV number in healthy control individuals compared to breast cancer patients with primary tumours in situ and those with metastases present. Figure S2: A standard curve for 1 × 10^−8^ to 1 × 10^−14^ mol dilutions of synthetic miR-451a was reproduced, and a linear equation (A) (y = −3.70(×) − 14.80) for the murine study and (B) (y = −3.24 (×) − 11.31) for human sample analysis was accepted.
