# Supplementary material for: Investigating the Potential and Pitfalls of EV-Encapsulated MicroRNAs as Circulating Biomarkers of Breast Cancer
Source: Cells. 2020 Jan 7;9(1):141. doi: 10.3390/cells9010141 (PMC7016709; doi:10.3390/cells9010141)
Supplement: Supplementary file 1 [file cells-09-00141-s001.pdf]

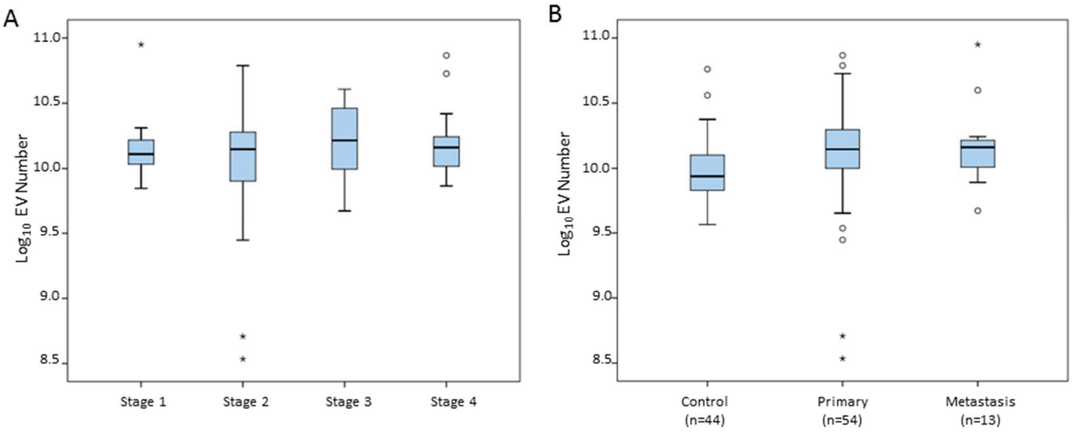

Supplementary Figure 1. (**A**) Investigation of relationship between circulating EV number and disease stage (**B**) Circulating EV number in healthy control individuals compared to breast cancer patients with primary tumours in situ and those with metastases present.

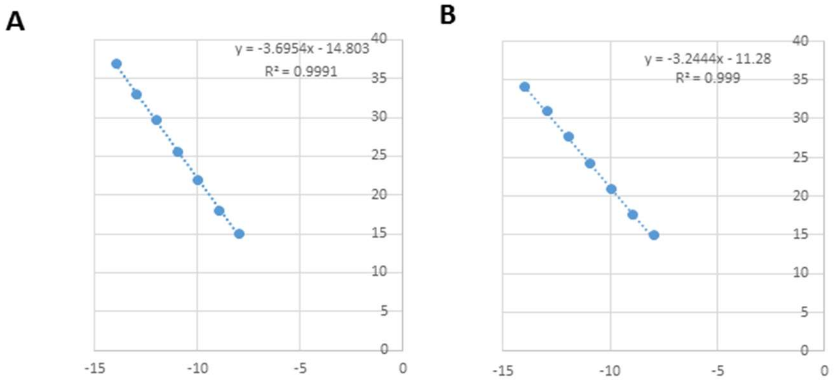

Supplementary Figure 2. A standard curve for 1 × 10^{-8} to 1 × 10^{-14} mol dilutions of synthetic miR-451a was reproduced and a linear equation (**A**) (y = -3.70 X–14.80) for the murine study and (**B**) (y = -3.24 X–11.31) for human sample analysis was accepted.
